# Nursing Unit Design, Nursing Staff Communication Networks, and Patient Falls: Are They Related?

**DOI:** 10.1177/1937586718779223

**Published:** 2018-06-19

**Authors:** Barbara B. Brewer, Kathleen M. Carley, Marge Benham-Hutchins, Judith A. Effken, Jeffrey Reminga

**Affiliations:** 1 The University of Arizona, Tucson, AZ, USA; 2 Carnegie Mellon University, Pittsburgh, PA, USA; 3 The University of Texas at Austin, Austin, TX, USA

**Keywords:** nursing unit design, social network analysis, communication structure, patient falls

## Abstract

**Purpose:**

The purpose of this research is to (1) investigate the impact of nursing unit design on nursing staff communication patterns and, ultimately, on patient falls in acute care nursing units; and (2) evaluate whether differences in fall rates, if found, were associated with the nursing unit physical structure (shape) or size.

**Background:**

Nursing staff communication and nursing unit design are frequently linked to patient safety outcomes, yet little is known about the impact of specific nursing unit designs on nursing communication patterns that might affect patient falls.

**Method:**

An exploratory longitudinal correlational design was used to measure nursing unit communication structures using social network analysis techniques. Data were collected 4 times over a 7-month period. Floor plans were used to determine nursing unit design. Fall rates were provided by hospital coordinators.

**Results:**

An analysis of covariance controlling for hospitals resulted in a statistically significant interaction of unit shape and size (number of beds). The interaction occurred when medium- and large-sized racetrack-shaped units intersected with medium- and large-sized cross-shaped units.

**Conclusion:**

The results suggest that nursing unit design shape impacts nursing communication patterns, and the interaction of shape and size may impact patient falls. How those communication patterns affect patient falls should be considered when planning hospital construction of nursing care units.

For years, operational efficiency has been a chief hospital objective and a fundamental consideration in the design of nursing units. Factors such as the size of patient rooms and their positioning within the nursing unit were understood to influence a unit’s shape and its operational efficiency. More recently, designers found that, within a nursing unit, the number and placement of centralized or decentralized nursing workstations also influenced operational efficiency, that is, how far nurses must travel to provide care (Fay, Carll-White, Schadler, Isaacs, & Real, 2017; Kazanasmaz, & Tayfur, 2012). Whether these operational efficiency issues should also include nursing staff communication patterns remained unclear. However, Kalisch and Begeny (2005) found that the physical environment affected nursing team function; and when nurses were confronted with long or double corridors limiting their ability to see coworkers, they did not seek help when needed.

Concerns about patient safety (Stichler, 2017), a complex problem with no single or simple solutions, have also been addressed in hospital and room designs.

The results of numerous studies have suggested that visibility (observability) of patients from nursing work areas, such as nurses’ stations and medication preparation areas, is important in improving safety outcomes such as patient falls (Hadi & Zimring, 2016; Hignett, 2010; Lopez, Gerling, Carey, & Kanak, 2010; Taylor & Hignett, 2016). Long corridors with greater space between rooms or between the two ends of the hall reduce visibility, and therefore the nursing staff’s ability to surveil their patients (Hadi & Zimring, 2016). Where visibility is low, work-arounds such as bed alarms and video monitors have been used, although inconsistently and with mixed results (Lopez et al., 2010). Because communication is one of the chief contributors to negative patient safety outcomes (Institute of Medicine, 2000), a possible reason for the mixed result with work-arounds is the failure to explore the impact of nurses’ communication patterns on patient safety and how those communication patterns may differ when constrained by different nursing unit shapes with varying levels of patient observability. Trzpuc and Martin (2010) used space syntax theory as a framework to understand relationships among spatial aspects of medical surgical nursing units on visibility and accessibility of staff and patients and found that both visibility and accessibility impacted staff communication. In a multimethod study conducted by Real, Bardach, and Bardach (2017), nursing staff reported that teamwork and communication with each other were reduced, while patient falls increased, in nursing units with decentralized nursing stations.

The current study was part of a larger research project in which staff communication patterns in 24 nursing units in three acute care hospitals were described using social network analysis (SNA) at four points in time over a 7-month period to assess their stability over time. Finding a set of stable metrics that could be used by organizations to assess communication was imperative if the data collected at a particular time would be useful to those organizations. Unlike other SNA environments, staff members in hospitals work 3 days a week, so the SNA environment may be very inconsistent. Assuming a stable set of metrics was found, a second goal was to explore the possible association of the stable communication metrics and the nursing unit physical structure (shape) with safety outcomes, specifically, fall rates. Usually, all nursing units within a hospital building have a similar shape. This was true in our sample too. However, because some nursing units in our sample were in different buildings within the same hospital, we also attempted to differentiate the impact of nursing unit shape differences versus characteristics common across all nursing units (despite their different physical designs) within a single hospital on fall rates.

## Method

### Sample and Setting

The sample for this study consisted of 24 nursing units from three acute care hospitals in the southwestern United States. Individual-level data from 1,561 nursing unit staff were collected and then aggregated using group means to represent their nursing units, resulting in a nursing unit sample size of 24. Most (66%) of the individual nursing staff sample was comprised of registered nurses (RNs), with patient care technicians (PCTs) making up the second largest group (27%). Unit clerks (UCs) and other assistive personnel such as monitor watchers who were working on the days of data collection comprised the remaining 7% of the sample. Network data were collected via questionnaire from nursing unit staff over four 24-hr periods, which will be described in more detail in the section on measures. Network data from the four data collections were aggregated to create information sharing and decision-making networks for each nursing unit. The average number of staff for the aggregated unit networks ranged from 9.75 to 35. Human subjects review of the study was performed at the universities of the principal investigators and each of the three hospitals before any data were collected.

The sample of nursing units (*N* = 24) included the following specialties: progressive care (*n* = 3), telemetry (*n* = 3), oncology (*n* = 5), neurology (*n* = 2), general medical (*n* = 2), orthopedic (*n* = 2), general surgical (*n* = 3), observation (*n* = 1), women (*n* = 2), and cardiac (*n* = 1). Unit size ranged from 12 to 51 beds. For analysis, unit size was categorized as small equals 1–20 beds (*n* = 7), medium equals 21–35 beds (*n* = 10), and large equals 36–51 beds (*n* = 7). Four unit shapes were identified from floor plans: compact circle, compact square, racetrack, and cross. Unit size by nursing unit shape ranged from compact circle with 36 beds (*n* = 2), compact square with 16–27 beds (*n* = 7), racetrack with 12–40 beds (*n* = 9), and cross with 25–51 beds (*n* = 6). Most patient rooms were private. On Unit 1 (cross shaped), which had the most falls, there were nine private and nine semiprivate rooms. Units 8 and 16, which had no falls, had only private rooms. All facilities used sitters to observe patients when clinical staff felt constant observation was necessary to prevent injury. All facilities had bed alarms and other forms of technology to warn staff of fall risk potential. All nursing units used mobile communication devices, such as Vocera. All units had a combination of centralized and decentralized documentation and communication stations. The compact square units had centralized documentation and communication stations but augmented their centralized stations with workstations on wheels.

### Measurement

#### Networks

As is typical in SNA (Effken, Gephart, Brewer, & Carley, 2013), to collect nursing staff data to define the networks, a questionnaire was used to ask the same three questions 4 times (baseline, 1, 4, and 7 months). The three questions asked staff about (1) the frequency with which they discussed patient care with others working on their unit during their current shift (resulting in an information sharing network), (2) the frequency they asked others on the unit for advice, and (3) the frequency of others asking them for advice (Questions 2 and 3 were combined to form the decision-making network). The questions were typical of those used in SNA research (e.g., Who among this group of potential colleagues do you ask for advice? How often do you interact with X, Y, Z?; Patterson et al., 2013; van Beek et al., 2011). Frequency of communication for network questions was measured via a 5-point scale ranging from *not at all* = 0, *rarely* = 1, *some* = 2, *a lot* = 3 to *constantly* = 4. Table 1 provides definitions of the metrics used to describe the resulting networks.

**Table 1.** Network Metrics Used in the Study With Their Definitions.

Network Metric	Definition
Node size	The number of nodes (in this case, individual staff) in the network.
Density	The percentage of actual to possible connections between nodes.
Weighted density	Density weighted by frequency of communication.
Total degree centrality	How many neighbors a node is connected to—includes both incoming (in-degree) and outgoing (out-degree) communication.
Betweenness centrality	Measures the number of times that connections must pass through a single individual to be connected (i.e., which person is most central to the network as a whole and likely to be the most influential with the most group knowledge). Higher scores describe organizations in whom many people play this central role.
Eigenvector centrality	Measure of node connections to highly connected people. A person well connected to well-connected people can spread information quickly and could be critical when rapid communication is needed.
Clustering coefficient	Extent to which there are small clusters (cliques). A higher clustering coefficient supports local information diffusion as well as a decentralized infrastructure because employees are likely to share information and know what is happening in their work group.
Average distance	The average number of connections along the shortest paths for all possible pairs of network nodes. Average distance provides a measure of information efficiency.
Diffusion	The speed with which information can travel through the network.

#### Patient fall rate

Patient fall rates were provided by each hospital for each nursing unit in the study for each of the 4 months of data collection. To control for differences in unit size, patient fall rates were standardized as total patient falls per 1,000 patient days. To adjust for month-to-month variation of fall rates, a mean of the four monthly rates was calculated and used as an aggregate value in the analysis.

#### Nursing unit designs

**Figure 1.**
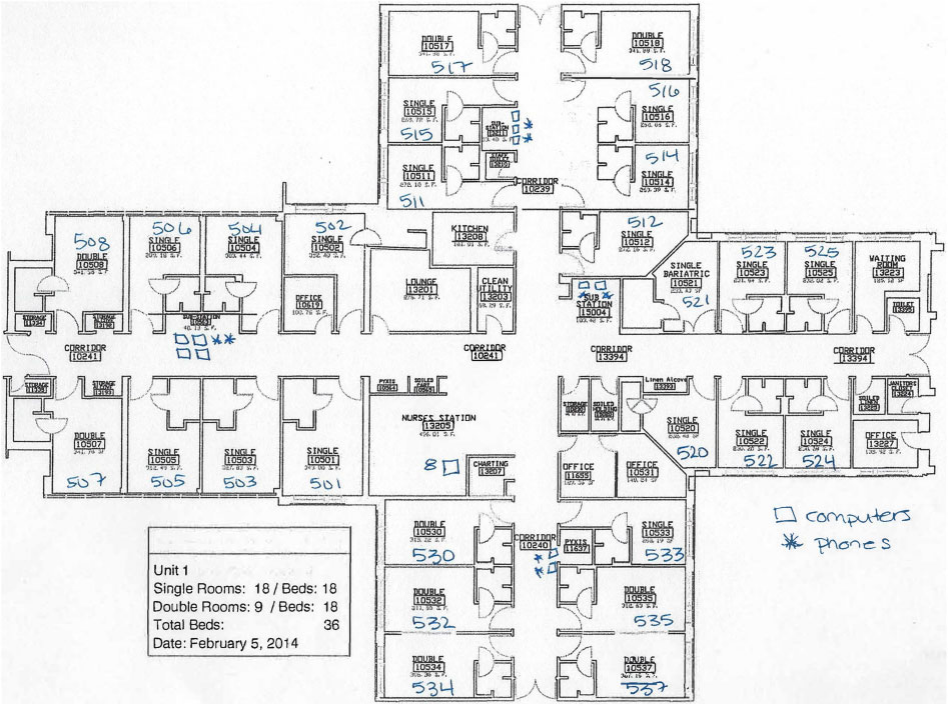
Floor plan for decentralized cross-shaped nursing unit (Unit 1). Open squares marked on the floor plan denote computers and asterisks denote telephones.

**Figure 2. fig2-1937586718779223:**
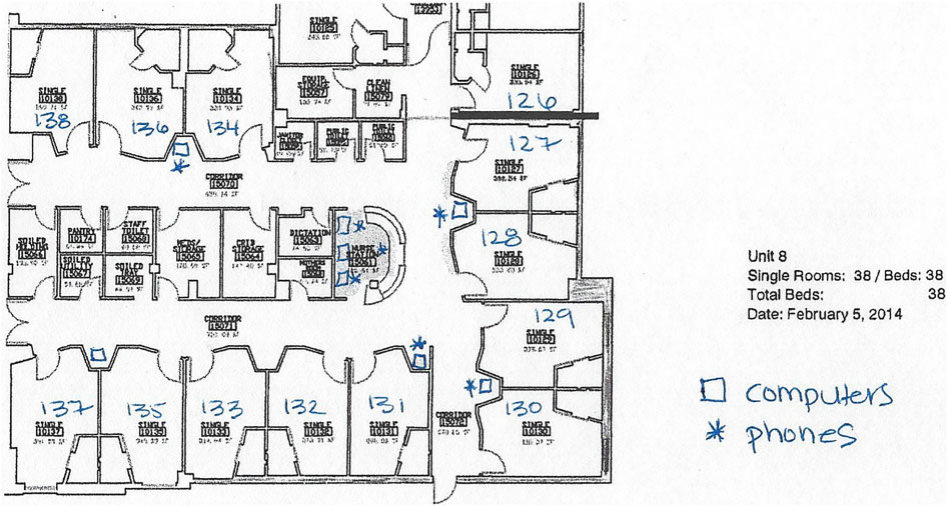
Floor plan for hybrid racetrack-shaped nursing unit (Unit 8). Open squares marked on the floor plan denote computers and asterisks denote telephones.

We obtained floor plans for each of the 24 nursing units studied. Two of the authors independently compared the floor plans with six standard unit design shapes (Bobrow & Thomas, 2000) and unanimously agreed that our sample comprised four of the six types (cross, racetrack, compact circle, and compact square). Some hospitals had multiple buildings. The nursing unit shapes were generally consistent within a building, but not always consistent within a hospital. All nursing units in the study had both centralized and decentralized work stations located outside of patient rooms. Some of the units with centralized workstations (nursing stations) used mobile workstations to provide access to computers away from the central workstation. Figures 1 [Fig fig2-1937586718779223]–3 depict the floor plans of the nursing units with the fewest and greatest number of falls. When examining differences in the fewest and greatest patient fall rates across all shaped units, differences were noted. The compact square- (Unit 16) and racetrack (Unit 8)-shaped units did not have any patient falls. The cross-shaped unit (Unit 1) had the greatest number of falls. There were also differences among these different shaped units with the distribution of nurses’ work spaces. Further classification of unit characteristics (following the terminology used by Hua, Becker, Wurmser, Bliss-Holtz, and Hedges, 2012) revealed that the compact square-shaped unit had a centralized nurses station, the racetrack-shaped unit had a hybrid design with a centralized nurses station and decentralized touchdown areas (small workstations containing a computer and phone) outside patient rooms throughout the nursing unit, and the cross-shaped unit had a decentralized design with a small centralized nurses station and several small substations or pods located throughout the unit.

**Figure 3.**
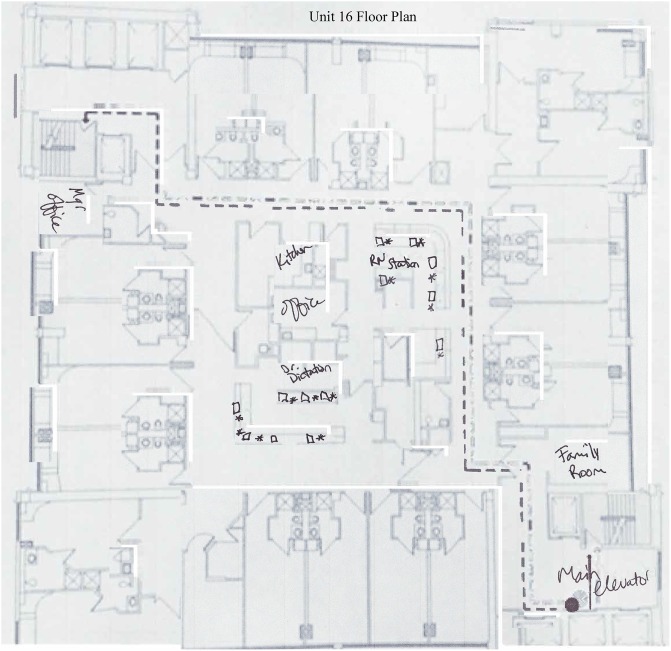
Floor plan for centralized compact square-shaped nursing unit (Unit 16). Open squares marked on the floor plan denote computers and asterisks denote telephones. Two mobile workstations available for staff use not depicted in the drawing.

#### Data collection and analysis

As noted earlier, nursing communication data were measured at baseline and 1, 4, and 7 months later. In addition to the three network questions discussed earlier, staff were asked about their confidence in the information they received, their demographics (e.g., job and experience), and whether the day of data collection was normal (typical) or not. Patient falls occurred throughout the entire month that data were collected and not necessarily on the day of data collection, because staff data collection and patient falls occurred during a single month, but not necessarily on a day a fall occurred, we did not include staff data related to workload variance in the current study.

#### Technology used for data collection

All staff communication data were collected at the end of their shifts (both day and night shifts for the same 24-hr period) using a handheld tablet with wireless Internet connection (for details, see Benham-Hutchins, Brewer, Carley, Kowalchuk, & Effken, 2017). The handheld tablet contained an electronic version of the staff survey. Using a handheld tablet and associated website, rather than paper and pencil, allowed a more efficient method for the research team to list only the staff working on the nursing unit that day, thus making the survey less onerous for staff (it took approximately 15 min or less to complete).

Nursing staff responses were uploaded from the individual handheld devices to a website where they were automatically converted into the format required for ORA (3.0.9x) (Carley, 2018), a network analysis software application, which was used for network analysis. ORA was used to construct a matrix that denoted, for each staff member, those nursing staff members with whom they had discussed patient care (Question 1) during their just-completed shift. The resulting matrix defines the “information-sharing network.” The network for each nursing unit was viewed visually and metrics generated to describe its characteristics. The “advice network” was derived in similar fashion, but since two questions were used (*How often did you go to any of these coworkers for patient care–related decision-making advice? How often did any of these coworkers come to you for patient care–related decision-making advice?*), there were initially two separate networks which were then merged into one.

Network analysis produces a set of commonly accepted metrics for each network measuring such characteristics as nodes (number of nursing staff) density of communication, weighted density (weighted by frequency of communication), centrality, small groups (clustering), average distance (measures efficiency of communication), and speed of communication (diffusion). Nine of these metrics (listed in Table 2) were found to be stable (within standard deviation [*SD*] = 0.5) over the 4 time periods and, therefore, were used for all further analyses.

**Table 2.** Mean (*M*), Standard Deviation (*SD*), and Analysis of Variance (ANOVA) of Network Metrics by Unit Shape.^a^

Network Metric^b^	Compact Circle^c^ (*n* = 2)		Compact Square (*n* = 7)		Race Track (*n* = 9)		Cross^d^ (*n* = 6)		ANOVA Result (*df* = 3)
	*M*	*SD*	*M*	*SD*	*M*	*SD*	*M*	*SD*	
Node size^e^	24.25	3.53	15.29	1.88	16.19	5.13	26.33	6.40	*F* = 8.08, *p* = .001
Density^e^	0.35	0.07	0.40	0.04	0.43	0.06	0.30	0.08	*F* = 5.07, *p* < .01
Weighted density^e^	0.21	0.07	0.26	0.03	0.27	0.05	0.17	0.05	*F* = 6.65, *p* < .01
Total degree centrality^e^	0.22	0.07	0.27	0.03	0.28	0.05	0.29	0.05	*F* = 6.74, *p* < .01
Betweenness centrality^f^	0.02	0.00	0.03	0.00	0.05	0.01	0.03	0.00	*F* = 9.56, *p* < .001
Eigenvector centrality^e^	0.26	0.02	0.34	0.02	0.35	0.06	0.26	0.04	*F* = 6.99, *p* < .01
Clustering coefficient^e^	0.50	0.06	0.53	0.04	0.50	0.04	0.42	0.05	*F* = 7.92, *p* = .001
Average distance^g^	2.68	0.66	2.28	0.18	2.60	0.27	2.99	0.31	*F* = 6.47, *p* < .01
Diffusion^h^	0.71	0.05	0.67	0.05	0.87	0.12	0.77	0.03	*F* = 9.31, *p* < .001

^a^ Note that *n* for each unit shape varies as shown in the table. ^b^With two exceptions (node size and average distance) that are actual counts, metrics are measured on a 0–1 scale. ^c^Two compact circles with short connecting corridor containing elevators and support space. ^d^In one unit, one arm was shorter than the others. ^e^Cross different from compact square and racetrack. ^f^Compact circle different from racetrack and cross, compact square different from racetrack. ^g^Cross different from compact square. ^h^Compact square different from racetrack.

SPSS (IBM SPSS Statistics) Version 24 was used to analyze the data. Analysis of variance was used to evaluate network metrics (Table 2); and analysis of covariance (ANCOVA) was used to evaluate fall rates for differences based on nursing unit design and size (number of beds) while controlling for variance common to a hospital.

## Results

Results for network metric differences across all 24 units will be reported first, followed by results for the three units with the highest and lowest fall rates. Table 2 summarizes differences in network metrics averaged over the four data collection periods for the 24 units. Although the racetrack had one of the lowest node counts (nursing staff), it had the highest communication density and weighted density. The racetrack also had the fastest (87%) diffusion rate and the second shortest average distance, which measures efficiency of the communication network. Distance is a measure of the number of links (connection between two people) on the shortest path between two nodes. Because two people may be connected through others and not directly with each other, the number of links will vary. The smaller the average distance value, the more efficient the communication in the network. The racetrack had high centrality (i.e., many links to highly connected people). The cross-shaped nursing units had the highest node counts, the lowest density, moderate centrality, and the greatest average distance. Cross-shaped nursing units had significantly more patient beds (*M* = 37.3, *SD* = 11.67) than compact square- (*M* = 21.9, *SD* = 4.14) or racetrack (*M* = 23, *SD* = 9.5)-shaped nursing units, *F*(3, 20) = 5.0, *p* = .01. Each of the two compact circle-shaped nursing units contained 36 beds.

**Figure 4.**
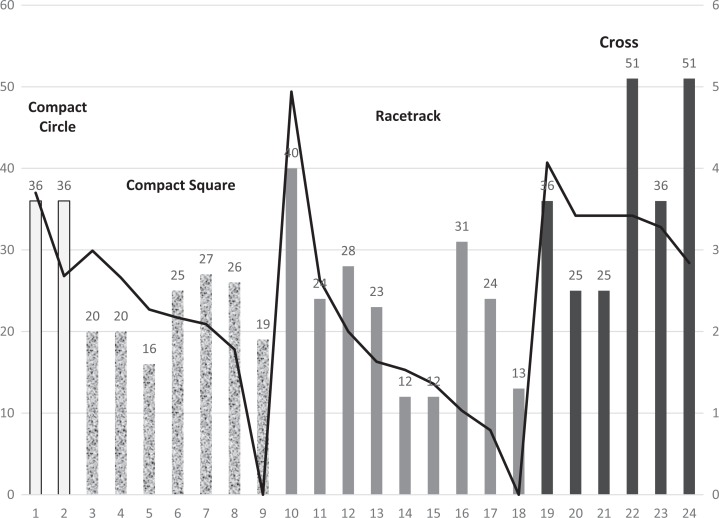
Graphic illustration of fall rates (line) and unit shapes and bed size represented by height of bars. The *y*-axis scale on left represents bed size and on right represents fall rate. Bars along *x*-axis are shaded to represent nursing unit shapes.

**Figure 5.**
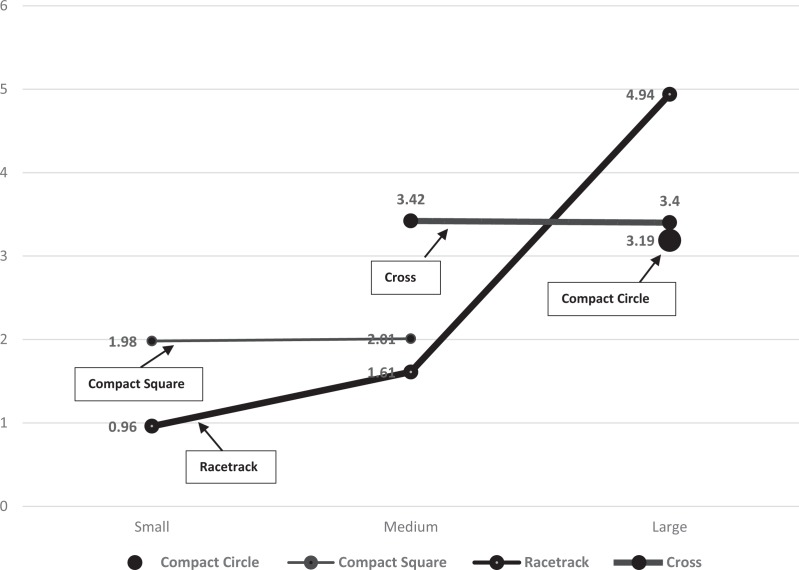
Graphic representation of interaction between medium- and large-sized racetrack and medium- and large-sized cross-shaped nursing units. The *y*-axis scale represents fall rate and *x*-axis represents small- (1–20 beds), medium- (21–35 beds), and large-sized (36–51 beds) units.

Average falls per 1,000 patient days ranged from 0 to 4.94, with a mean fall rate of 2.4 (*SD* = 1.2). To control for the differences in fall rates related to hospital characteristics unrelated to nursing unit shape or size, an ANCOVA was performed with hospital as a covariate and nursing unit shape and size as main effects. The model revealed a statistically significant interaction of nursing unit shape and size, *F*(2, 15) = 6.317, *p* = .01. Figure 4 illustrates the relationships among nursing unit shape, bed size, and fall rate. The primary *y*-axis represents bed size and the secondary *y*-axis represents fall rate. Bars are grouped according to nursing unit shape in the following order from left to right, compact circle (*n* = 2), compact square (*n* = 7), racetrack (*n* = 9), and cross (*n* = 6). Note that cross-shaped nursing units are generally larger and have more falls per 1,000 patient days than similarly sized units. Figure 5 illustrates the interaction between cross-shaped nursing units and racetrack-shaped units (when limited to those either medium or large sized).

**Figure 6.**
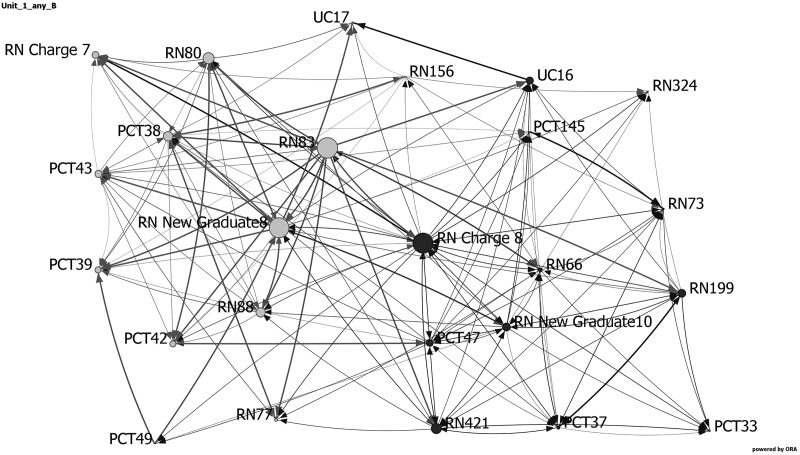
Network visualization for decentralized cross-shaped nursing unit (Unit 1) at baseline, showing the day and night shifts as light gray and black nodes, respectively. Node size depicts relative eigenvector centrality values for each staff member. Staff roles are identified by node names: registered nurses (RN), patient care technicians (PCT), unit clerk (UC), and charge nurse (RN charge), followed by participant number. The width of the links between nodes indicates frequency of communication, with darker, wider lines representing more frequent communications. Arrows depict direction of communication.

**Figure 7. fig7-1937586718779223:**
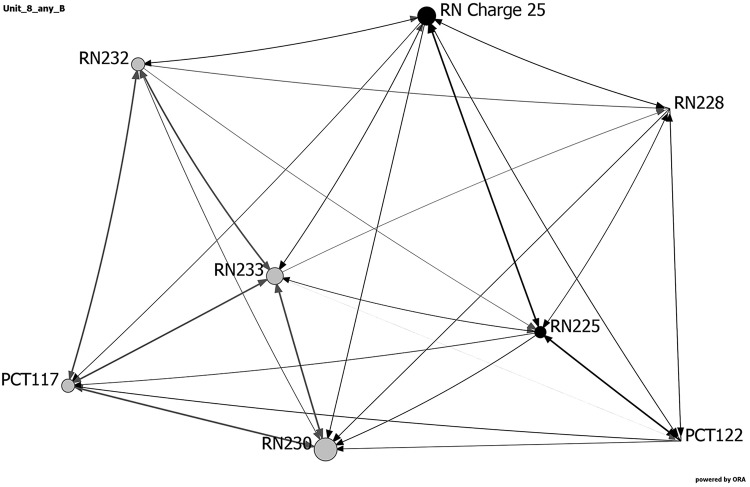
Network visualization for hybrid racetrack-shaped nursing unit (Unit 8) at baseline, showing the day and night shifts as light gray and black nodes, respectively. Node size depicts relative eigenvector centrality values for each staff member. Staff roles are identified by node names: registered nurses (RN), patient care technicians (PCT), unit clerk (UC), and charge nurse (RN charge), followed by participant number. The width of the links between nodes indicates frequency of communication, with darker, wider lines representing more frequent communications. Arrows depict direction of communication.

Table 3 and Figures 6 [Fig fig7-1937586718779223]–8 show the network metrics and visualizations for the three nursing units with the highest and lowest fall rates. Visualizations depict the three units at baseline, showing the day and night shifts as light gray and black nodes, respectively. Node size depicts relative eigenvector centrality values for each staff member. Staff roles are identified by node names: RNs, PCTs, UCs, and charge nurse (RN charge), followed by participant number. The width of the links between nodes indicates frequency of communication, with darker, wider lines representing more frequent communications. Arrows depict direction of communication.

**Table 3.** Network Metrics for Three Units With Highest and Lowest Fall Rates.

Unit	Node Size	Diffusion	Average Distance	Density	Weighted Density	Total Degree Centrality	Betweenness Centrality	Eigenvector Centrality	Clustering Coefficient
1	25	.74	.69	.29	.17	.18	.03	.26	.42
8	9	.75	.45	.51	.37	.39	.04	.39	.55
16	17	.81	.57	.5	.3	.31	.03	.33	.59

*Note*. Units 8 (hybrid racetrack) and 16 (centralized compact square) had no falls. Unit 1 (decentralized cross) had an average rate of 4.94 falls per 1,000 patient days.

**Figure 8.**
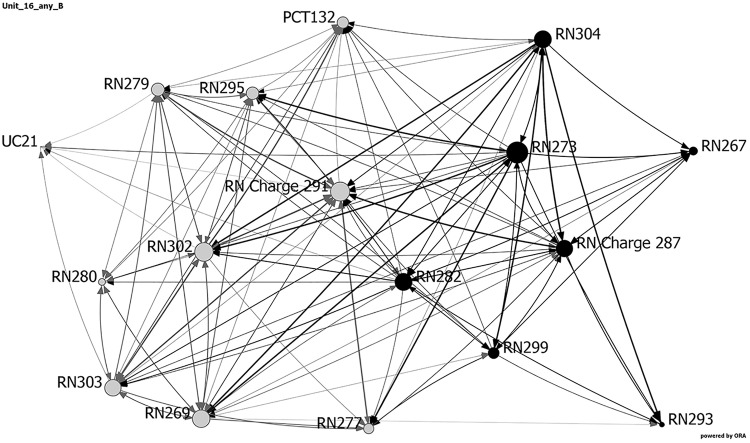
Network visualization for centralized compact square-shaped nursing unit (Unit 16) at baseline, showing the day and night shifts as light gray and black nodes, respectively. Node size depicts relative eigenvector centrality values for each staff member. Staff roles are identified by node names: registered nurses (RN), patient care technicians (PCT), unit clerk (UC), and charge nurse (RN charge), followed by participant number. The width of the links between nodes indicates frequency of communication, with darker, wider lines representing more frequent communications. Arrows depict direction of communication.

As noted previously, two nursing units (one a hybrid racetrack and the other a centralized compact square) reported no falls during the 4 months data were collected. These two units had faster diffusion, shorter average distances, higher density and weighted density, more clustering, and a higher percentage of staff who were central to the network. The decentralized cross-shaped nursing unit with the most falls had more staff (higher node size), fewer connections between staff (lower density), higher average distance, a lower percentage of central (influential) individuals, and fewer clusters (small groups).

## Discussion

The results in this study support those from other studies (Fay et al., 2017; Real, Barduch, & Barduch, 2017). It appears that nursing unit design and size (particularly if medium or large units) may affect fall rates because some unit design shapes allow less close visual or aural monitoring than others, which is crucial for preventing falls. Further research will be needed to validate this conclusion. With the exception of instances where nursing units were located in different wings or clinical areas, the units in each of the three study site hospitals shared a single design shape unique to that hospital. It is uncertain how other hospital variables such as safety cultures or unit cultures that were not measured directly could have affected the communication network findings.

A second finding of this study was that network communication metrics differed with nursing unit shape. In general, cross-shaped decentralized units had less effective communication structures (as evidenced by lower density, diffusion, clustering coefficient, and eigenvector centrality network metrics) than racetrack-shaped hybrid (a centralized nurses workstation with decentralized touchdown areas) or compact square-shaped centralized units. This result may reflect lower visibility or access to other staff because of the longer straight corridors associated with the cross-shaped units in this study. This finding is similar to those reported by Fay, Carll-White, Schadler, Isaacs, and Real (2017); Real et al. (2017); and Kalisch and Begeny (2005), who found that visibility of other staff affected communication with each other, and decentralized workstations resulted in less access and less communication. This finding was also similar to that of Hua, Becker, Wurmser, Bliss-Holtz, and Hedges (2012), who found that a move from a decentralized design to a hybrid design resulted in nurses feeling more isolated with fewer social interactions (i.e., communication).

Because of the small sample size of nursing units in the current study, we must be cautious in generalizing the results. However, if these results do apply broadly, hospital designers might do well to ensure that nursing staff visualization of patients is maximized to reduce fall rates, one of the more expensive complications for patients and hospitals. New technologies, such as computer simulation, can include consideration of nurses’ work flow and distances traveled for various activities (e.g., assessment, medication administration, rounding, and handoffs) as well as the more traditional data such as room size, capacity, equipment, and floor plan (O’Hara, 2014).

Researchers (Pati, Evans, Harvey, & Bazuin, 2012a; Pati, Harvey, & Thurston, 2012b) have used computer simulation to assess the impact of decentralizing nursing support spaces on nurses’ walking distances and use of time. The results of this study suggest that communication network data may also be an important consideration to support desirable communication patterns that have the potential to reduce the number of patient falls. As Rashid (2015) notes:

Unit layouts can do a great deal to encourage communication and collaboration by eliminating visual and physical barriers. Conversely, they can all too easily disrupt existing relationships by imposing unnecessary barriers. The identity of a practice team may depend on how visible the members are to the patient and patient family within a unit. It may also depend on how the members are visible to each other within the unit. (p. 632)

In the current study, the specific unit design shapes that were shown to support better communication and reduce falls also increased bed visibility. A mathematical technique, targeted visibility index (TVI; Lu, 2010; Lu & Zimring, 2012), has been proposed as a technique to measure patient bed visibility in nursing units. These authors used TVI to compare various unit shapes and concluded that radial and double corridor units had the best visibility. However, as Rashid (2015) noted, TVI methodology only uses geometry and omits the human factors involved. Space Syntax analysis is another technique that could be useful to identify sight lines, as well as travel paths, under different unit designs, but may have some of the same limitations.

Some researchers have also linked unit designs with low-visibility rooms to patient mortality. One study of 664 patients admitted to the Columbia University Medical Center in 2008 concluded that patient mortality was higher (82%) in low-visibility rooms than in high-visibility rooms (64%; Leaf, Homel, & Factor, 2010). Lu, Ossmann, Leaf, and Factor (2014) reanalyzed Leaf et al.’s (2010) data using TVI and reported that over 35% of the difference in mortality for the sickest of these patients was consistent with low visibility. While our current study used SNA to evaluate the nursing unit staff communication structure and the relationships among communication structural characteristics (i.e., metrics) and nursing unit shapes and size to patient falls, we did not examine patient mortality. Further research incorporating space syntax theory as a framework along with SNA may add further context to understanding the impact of the structural layout of the physical environment on nursing staff communication and patient outcomes.

The current study did not examine the effect of overall nursing unit patient acuity levels or nursing staff experience levels or tenure with the hospital, which could have also influenced the communication patterns among nurses. Less experienced nurses may have sought more advice (advice network) or nursing staff on units with higher average patient acuity levels may have discussed patient care more frequently (information sharing network).

## Conclusion

This exploratory study examined the association of nursing staff communication network metrics with nursing unit design shapes and patient falls. Although our sample was small (24 nursing units), finding a link between the communication patterns consistent with specific unit design shapes and sizes and patient falls seems to support other studies reported in the literature linking visibility with patient falls. More research is needed, but the results of this study further emphasize the need for hospital designers to work closely with nursing staff to determine, not only the most efficient and esthetically beautiful designs but also those designs that maximize patient safety.

***This exploratory study examined the association of nursing staff communication network metrics with nursing unit design shapes and patient falls. Although our sample was small (24 nursing units), finding a link between the communication patterns consistent with specific unit design shapes and sizes and patient falls seems to support other studies reported in the literature linking visibility with patient falls***.

***… the results of this study further emphasize the need for hospital designers to work closely with nursing staff to determine, not only the most efficient and esthetically beautiful designs but also those designs that maximize patient safety***.

## Implications for Practice

- Specific nursing communication patterns measured by network analysis metrics may be linked to specific unit design shapes, particularly those that affect patient and nurse visibility, and can affect the frequency of patient falls.
- Hospital designers should work with nursing staff to determine not only the most efficient and esthetically beautiful designs but also those designs that maximize patient safety.

## Supplemental Material

Supplemental Material, Nursing_Unit_Design,_Nursing_Staff_Communication_Networks,_and_Patient_Falls_Are_They_Related - Nursing Unit Design, Nursing Staff Communication Networks, and Patient Falls: Are They Related? Click here for additional data file. Supplemental Material, Nursing_Unit_Design,_Nursing_Staff_Communication_Networks,_and_Patient_Falls_Are_They_Related for Nursing Unit Design, Nursing Staff Communication Networks, and Patient Falls: Are They Related? by Barbara B. Brewer, Kathleen M. Carley, Marge Benham-Hutchins, Judith A. Effken, and Jeffrey Reminga in HERD: Health Environments Research & Design Journal
