# Supplementary material for: Nursing Unit Design, Nursing Staff Communication Networks, and Patient Falls: Are They Related?
Source: HERD. 2018 Jun 19;11(4):82–94. doi: 10.1177/1937586718779223 (PMC6236589; doi:10.1177/1937586718779223)
Supplement: Supplemental Material, Nursing_Unit_Design,_Nursing_Staff_Communication_Networks,_and_Patient_Falls_Are_They_Related - Nursing Unit Design, Nursing Staff Communication Networks, and Patient Falls: Are They Related? [file Nursing_Unit.pdf]

Nursing Unit Design, Nursing Staff Communication networks, and Patient Falls: Are They Related?
Authors: Brewer, B.B., Carley, K.M., Benham-Hutchins, M., Effken, J.A., Reminga, J.
Course ID # HERD 68

Full Name	AIA # / EDAC #	Email	Phone #

Learning Objective 1: Be able to describe nursing communication patterns associated with safer (fewer falls) nursing unit designs.
Learning Objective 2: Be able to describe communication patterns associated with four different unit shapes.
Learning Objectives 3: Be able to identify design opportunities for improving communication among nursing staff.
Learning Objective 4: Be able to list communication metrics associated with more efficient communication.

Question 1: Nursing units that were decentralized and cross-shaped in this study had communication structures that were:

a. Less effective because they exhibited lower density, less diffusion, and lower clustering coefficients.
b. More effective because they exhibited higher density, faster diffusion, and more clustering.
c. Less efficient because they had a central nurses station.
d. More efficient because they had many touchdown areas for documentation.

Question 2: Racetrack-shaped hybrid units had the greatest number of staff?

a. True
b. False

Question 3: The unit shape with the fewest patient falls had:

a. Greater bed visibility
b. Slower diffusion of communication
c. Greater network density
d. Fewer individuals connected highly connected individuals

To earn AIA continuing education units please complete form, and email Catherine Ancheta at cancheta@healthdesign.org and your credits will be uploaded to your AIA account. To earn EDAC continuing education units please complete the form, retain the form for your records and self-submit the credits at the time of your EDAC renewal. The form is your “proof of attendance.”
